# Analysis of Tax sequence and its nucleotide polymorphisms in HTLV-1 cases from Argentina

**DOI:** 10.1186/1742-4690-12-S1-P65

**Published:** 2015-08-28

**Authors:** Jimena Salido, Maria E Eirin, Camila Cánepa, Gabriela Pataccini, Matías Ruggieri, Sindy Fraile, Mauricio Carobene, Mirna M Biglione, Carolina Berini

**Affiliations:** 1 Instituto de Investigaciones Biomédicas en Retrovirus y SIDA (INBIRS), UBA-CONICET, Buenos Aires, Argentina; 2 Laboratorio de Tuberculosis Bovina, Instituto de Biotecnología, CICVyA, INTA-Castelar, Buenos Aires, Argentina

## 

HTLV-1 is the etiologic agent of Adult T-cell Leukemia/Lymphoma (ATLL) and HTLV-1 Associated Mielopathy/Tropical Spastic Paraparesis (HAM/TSP). Tax, a critical viral factor for genomic activation and viral gene expression, has been implied in cell transformation. The aim of this study was to analyze the presence of nucleotide polymorphisms in the tax gene of HTLV-1 positive cases from different populations of Argentina. A total of 85 samples were analyzed: 10 from Kollas from Jujuy and 75 from residents of Buenos Aires province (BA) which included asymptomatic individuals and 21 hematology and neurology patients. DNA was obtained from PBMCs and tax sequence amplified by hemi-nested-PCR (1058pb), sequenced, edited and aligned. ATK-1 genome was used as reference sequence. These nucleotide sequences were then translated into amino acids, and thus, non-synonymous mutations were identified in the proteins´ functional domains. Punctual mutations were detected in all sequences. Regarding t he samples from BA, the mean nucleotide change was 6.2 ± 1.5 in asymptomatic carriers and 6.9 ± 2 in ATLL and HAM/TSP individuals (p>0.05). Mutations were detected in the nuclear localization, LZR, NF-kB dimerization and activation domains and nuclear export signal. These changes were detected both in asymptomatic carriers and patients (p>0.05). For the Kollas samples, the mean change variation was of 5.2 ± 0.5, similar to asymptomatic carriers, but lower than ATLL and HAM/TSP individuals from BA (p>0,05). Two polymorphisms (T7914C and C7982T) were found in sequences from Jujuy but absent in sequences from BA. Considering the functional domains, two variations were present in all 10 sequences, one in the NF-kB dimerization and activation domain (A221V) and the other with an unknown function (F304N), that were also detected in residents from BA. These results corroborate the presence of different polymorphisms in the tax sequence but no association among any particular mutation and the pathologies was established.

